# Biogeographical Patterns of Herbivore Arthropods Associated with *Chenopodium quinoa* Grown along the Latitudinal Gradient of Chile

**DOI:** 10.3390/plants10122811

**Published:** 2021-12-19

**Authors:** Rodrigo A. Chorbadjian, María I. Ahumada, Francisco Urra, Mario Elgueta, Todd M. Gilligan

**Affiliations:** 1 Departamento de Ciencias Vegetales, Facultad de Agronomía e Ingeniería Forestal, Pontificia Universidad Católica de Chile, Santiago 7820436, Chile; miahumad@uc.cl; 2 Sección Entomología, Museo Nacional de Historia Natural, Casilla 787, Santiago 7820436, Chile; francisco.urra@mnhn.gob.cl (F.U.); Mario.Elgueta@mnhn.cl (M.E.); 3 USDA-APHIS-PPQ-Science and Technology, Identification Technology Program, Fort Collins, CO 80526, USA; todd.m.gilligan@usda.gov

**Keywords:** insects, pests, quinoa, distribution, Chile

## Abstract

Identifying the particular guilds of herbivore arthropods that affect the production of crops is key to developing sustainable pest-management strategies; however, there is incomplete information about the identity of herbivore arthropods that could potentially damage the production of both highland and lowland quinoa landraces grown in Chile. By both reviewing the literature and conducting field collections across a large latitudinal gradient, we generated an updated list of 43 herbivore arthropods associated with quinoa production in Chile. In general, most species are polyphagous feeders, and only seven are specialists. The number and identity of species varied in relation with the latitude, such that four distinctive assemblages of herbivores were identified, each containing 32, 27, 34, and 22 species between latitudes 18–26, 26–32, 32–40, and 40–44° S, respectively. The most northern production area (18–26° S) is affected by nine unique species, including the major quinoa pest *Eurysacca quinoae* Povolný (Lepidoptera: Gelechiidae). Similarly, the central area (32–40° S) contains four unique species, including *Eurysacca media* Povolný (Lepidoptera: Gelechiidae) and *Orthotylus flavosparsus* (Sahlberg) (Hemiptera: Miridae). The particular species assemblages described here will help further development of local pest-management practices.

## 1. Introduction

Quinoa (*Chenopodium quinoa* Willd.) is an annual plant mainly grown to obtain grains for human consumption. The interest in cultivating this crop has increased in recent years due to its high nutritional value and its tolerance to soil salinity and drought stress [1,2,3]. Quinoa is cultivated over a wide variety of environments in South America, extending from the high altitudes (>3500 m above sea level (m.a.s.l.)) of the Andean Altiplano areas of Bolivia, Chile, and Perú, to the lowland/coastal areas of Chile and Perú [4,5]. Two distinctive quinoa ecotypes are cultivated in Chile, the salares and the coastal ecotypes [4]. Plants belonging to the salares ecotype grow in the northern region of Chile (18–29° S), which is separated from the central and southern regions (33–43° S) by the Atacama Desert and is agroecologically more similar to the Altiplanos of Perú and Bolivia. In the central and southern production regions, the coastal ecotype of quinoa is produced along with many other agricultural vegetables, crops, and fruit trees. Regarding the distribution of quinoa cultivation in Chile, the northern zone (17–26° S) accounted for 31%, the central zone (29–36° S) accounted for 64%, and the southernmost zone (37–44° S) accounted for 5% of total production during the years 2015–2016 [6]. 

The variety of agroecological environments over which quinoa is cultivated can influence the diversity of arthropods that negatively affect its production. For instance, while species of *Eurysacca* (quinoa moth, Lepidoptera: Gelechiidae) are more frequently indicated as a major pest in the highlands [5,7,8], other species such as thrips (*Frankliniella occidentalis* (Pergande) (Thysanoptera: Thripidae)), aphids (*Macrosiphum euphorbiae* (Thomas) (Hemiptera: Aphididae)), and leafminers (*Liriomyza huidobrensis* Blanchard (Diptera: Agromyzidae)) add to the array of pest species than can reach high populations in lowland areas of Perú [9].

Understanding the herbivore communities is essential to developing sustainable insect pest management strategies in quinoa production; however, specific studies reporting insect species that feed on quinoa in Chile are scarce and incomplete. For instance, the general compendium of insect pests of economic importance in Chile published by Artigas [10] reported only five insect species that use quinoa as a host plant. A few years later, Lamborot et al. [11] reported five Lepidoptera species found in quinoa grown in central Chile. Later, another compendium of arthropod species found in Chilean agricultural plants reported only eight taxa [12]. Finally, Logarzo et al. [13] reported the finding of one leafhopper species on quinoa in the Chilean Altiplano area. Clearly, this is a low number of species when compared with the potential 78 arthropod taxa reported by Cruces et al. [5] in a recent compilation of quinoa pests that included: 29 species of Lepidoptera, 22 Hemiptera, 16 Coleoptera, 4 Orthoptera, 3 Thysanoptera, 3 Diptera, and 1 Acari. 

Because many insect species previously reported to feed on quinoa in other countries are also present in Chile, we decided to review and update this information. The underlying hypothesis is that it will be possible to identify distinct clusters of herbivore arthropods along the latitudinal gradient where quinoas are cultivated in Chile. In addition, we expected to identify latitudinal patterns in host range use, feeding habit, and geographical origin of these arthropods. Hence, the objective of this work was to generate an updated list of arthropod species associated with quinoa production in Chile by both reviewing the literature and conducting field collections on quinoa fields across a latitudinal gradient in a variety of agroecosystems. To further characterize the community of herbivores, species were classified according to their geographical origin and host-range use (i.e., generalist or specialist).

## 2. Results

Bibliographical evidence, together with field collections conducted in this study, allowed us to construct an updated list of arthropod herbivores that feed on quinoa plants in Chile, resulting in a total of 43 arthropod taxa. Across all latitudes, Lepidoptera is represented with 20 taxa in 4 families (Coleophoridae, Crambidae, Gelechiidae, and Noctuidae), Hemiptera with 15 taxa in 8 families (Aphididae, Cicadellidae, Coreidae, Lygaeidae, Miridae, Pentatomidae, Rhopalidae, and Triozidae), Coleoptera with 3 taxa in 3 families (Chrysomelidae, Curculionidae, and Meloidae), Thysanoptera with 2 Thripidae species, 1 Diptera (Agromyzidae), 1 Orthoptera (Acrididae), and 1 mite species (Acari: Tetranychidae) (Table 1). 

During field collections, we found and identified 19 taxa. Species identified included, *Achyra similalis* (Guenée) (Lepidoptera: Crambidae), *Agrotis ipsilon* (Hufnagel) (Lepidoptera: Noctuidae), *Aphis craccivora* Koch (Hemiptera: Aphididae), *Eurysacca quinoae* Povolný (Lepidoptera: Gelechiidae), *Frankliniella occidentalis* (Thysanoptera: Thripidae), *Feltia subterranea* (Fabricius) (Lepidoptera: Noctuidae), *Helicoverpa atacamae* Hardwick (Lepidoptera: Noctuidae), *Helicoverpa gelotopoeon* (Dyar) (Lepidoptera: Noctuidae), *Liorhyssus lineatoventris* (Spinola) (Hemiptera: Rhopalidae), *Liriomyza huidobrensis* (Diptera: Agromyzidae), *Macrosiphum euphorbiae* (Hemiptera: Aphididae), *Myzus persicae* (Sulzer) (Hemiptera: Aphididae), *Orthotylus flavosparsus* (Sahlberg) (Hemiptera: Miridae), *Tetranychus urticae* Koch (Acari: Tetranychidae), *Trichocyphus rubricollis* (Blanchard) (Coleoptera: Curculionidae), and *Trichoplusia ni* (Hübner) (Lepidoptera: Noctuidae). Three taxa were included only at the level of genera, as there was uncertainty regarding their species names. Specifically, further research is needed to clarify species identity for *Epitrix* sp. (Coleoptera: Chrysomelidae), *Pseudomeloe* sp. (Coleoptera: Meloidae), and *Copitarsia* spp. (Lepidoptera: Noctuidae). Specimens of these three genera were collected in this study and saved for further taxonomic analyses. 

The number of individual taxonomic units collected on each locality was 9 in the region of Tarapacá (Ancovinto site, 19° S), 13 in the Metropolitan region (Santiago and Pirque sites, 33° S), 7 in the O’Higgins region (Cahuil and Pailimo sites, 34° S), and 2 in the Los Lagos Region (Ancud sites, 42° S) (Table 1). Lepidoptera species were particularly abundant at the Ancovinto site. The identity of the quinoa moth *Eurysacca quinoae* was confirmed based on male genitalia structures that corresponded with its original description; particularly, the parabasal processes with broadly rounded clavate tips, symmetrical lanceolate saccular processes with an acute tip, and a long and slender valve [28]. Two species of *Helicoverpa*, *H. atacamae* and *H. gelotopoeon*, were confirmed based on morphological structures in comparison with descriptions and illustrations in Hardwick [30]. In particular, the two can be separated by the length of the male valvae, the shape of the everted vesica, and differences in setae on the foretibia [30]. We also identified a new host record for *Trichocyphus rubricollis*. *Trichocyphus rubricollis* was originally described by Kuschel [31] as a variety of *T. formosus*, to later recognize them as different specific entities [32]; Although Lanteri [33] establishes the synonymy between both names, Elgueta and Marvaldi [23] consider both as valid species. A few adults of *T. rubricollis* were detected feeding on quinoa leaves, chewing from the external margin of the leaves towards the central vein. Other species detected in this area included *Copitarsia* sp., *Aphis craccivora*, *Macrosiphum euphorbiae*, *Liriomyza huidobrensis*, and *Pseudomeloe* sp. 

In the central area (Metropolitana and O’Higgins areas), specimens collected included: *Achyra similalis*, *Agrotis ipsilon*, *Aphis craccivora*, *Copitarsia* spp., *Epitrix* sp., *Feltia subterranea*, *Frankliniella occidentalis*, *Liorhyssus lineatoventris*, *Liriomyza huidobrensis*, *Macrosiphum euphorbiae*, *Myzus persicae*, *Orthotylus flavosparsus*, *Tetranychus urticae*, and *Trichoplusia ni*. Among these species, a complex of chinch bugs (*L. lineatoventris* and *O. flavosparsus*), *Copitarsia* spp., and *L. huidobrensis* were more frequently collected. Our results also provide new information for the distribution range of *O. flavosparsus*, as it was known to be present in Chile [22], but no information on its distribution range and host use had been reported. In turn, the southernmost area of Ancud is characterized by the presence of fewer herbivore species. Here, we only collected *Copitarsia* spp. and *M. euphorbiae*. While only a few specimens of *M. euphorbiae* were detected, *Copitarsia* caterpillars were found more often but still in low numbers. 

**Figure 1:**
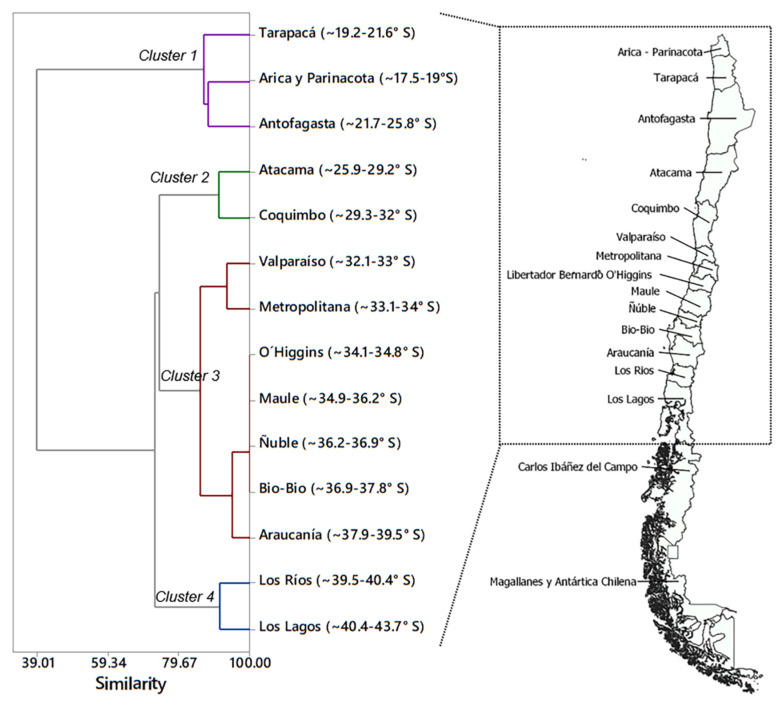
Dendrogram resulting from the multivariate cluster analysis of the geographical distribution variables of quinoa feeding arthropods present in Chile. Cluster description is presented in Table 2.

Regarding the total number of species expected to affect quinoa in Chile, we found that species assemblages varied in relation to the geographical region. Cluster analysis identified four groups with a percentage of similarity higher than 80% within each cluster (Figure 1). One cluster grouped the regions of Arica y Parinacota, Tarapacá, and Antofagasta (18–26° S) with 32 taxa; a second cluster included 27 taxa in the regions of Atacama and Coquimbo (26–32° S). In the central area of Chile, a third cluster included 34 taxa in the Regions of Valparaíso, Metropolitana, O’Higgins, Maule, Ñuble, Bio-Bío and Araucanía (32–40° S), and the fourth cluster assembled 22 taxa in the regions of Los Rios and Los Lagos (40–44° S) (Table 2). Interestingly, both Cluster 1 and 3 included unique species. Cluster 1 is defined by nine species that are uniquely found in the northernmost region, which is closest to the borders with Perú and Bolivia. This is the case for *Agrotis experta* (Walker) (Lepidoptera: Noctuidae), *Anacuerna centrolinea* (Melichar) (Hemiptera: Cicadellidae), *Chrysodeixis includens* (Walker) (Lepidoptera: Noctuidae), *Eurysacca quinoae, Pseudomeloe* sp., *Spodoptera frugiperda* (J.E. Smith) (Lepidoptera: Noctuidae)*, Spodoptera ochrea* (Hampson) (Lepidoptera: Noctuidae)*, Spoladea recurvalis* (Fabricius) (Lepidoptera: Crambidae), and *Trichocyphus rubricollis.* Similarly, Cluster 3 in the central region is uniquely defined by the presence of *Coleophora versurella* Zeller (Lepidoptera: Coleophoridae), *Epitrix* sp., *Eurysacca media* Povolný (Lepidoptera: Gelechiidae), and *Orthotylus flavosparsus*.

**Figure 2:**
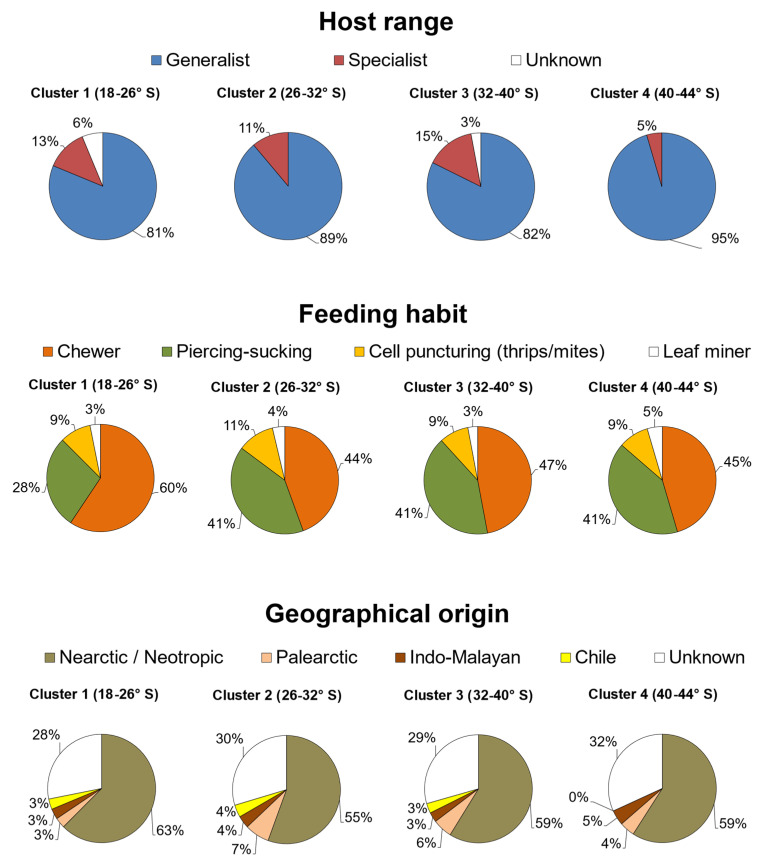
Latitudinal patterns of host range, feeding habit, and geographical origin of arthropod species that feed on quinoa in each latitudinal cluster of species.

In general, most species that feed on quinoa in Chile have a wide range of host use, given that 33 of them are polyphagous (77%) and only 7 are specialists (16%) (Table 1). When their geographical distribution is analyzed as a variable, the proportion of specialist herbivores ranges between 5 and 15% between parallels 18 and 39° S, with the lowest proportion in the southernmost area delimited by parallels 40–44° S (Figure 2). The distribution of the specialist herbivores *Eurysacca quinoae, E. media*, *Coleophora versurella*, and *Heterotrioza chenopodii* (Reuter) (Hemiptera: Triozidae) highly influenced this pattern due to their absence in the southern latitudes of Chile.

The feeding habit of the arthropod species that attack quinoa in Chile is mostly dominated by chewing and piercing–sucking insects. The relative proportion of chewing insects ranges between 44 and 60%, and that of piercing–sucking hemipterans between 28 and 41%, depending on the geographical area (Figure 2). Only three species puncture plant cells, including two thrips (*F. occidentalis* and *Thrips tabaci* Lindeman) and the two-spotted spider mite (*T. urticae*). The leafminer *L. huidobrensis* was the only species identified with this strict feeding habit, which is distributed across the entire Chilean territory (Table 1). *Eurysacca* species were classified as chewer insects, although it has been reported that they have a leaf-mining habit during the early stages of larval development [7].

Regarding the geographical origin of the 43 arthropod species reported to feed on quinoa in Chile (Table 1), 30 species are native to the New World, while only 2 are Palearctic, and 1 comes from the Indo-Malayan realm. Interestingly, *Oncopeltus miles* (Blanchard) (Hemiptera: Lygaeidae) is the only native species reported to feed on quinoa in Chile [10]. In turn, 10 species have an uncertain geographical origin, as these are cosmopolitan agricultural pests (Table 1).

## 3. Discussion

This is the first study to compile a list of arthropods that feed on quinoa in Chile, which will help quinoa growers and future insect–plant interaction research. Until this study, only 10 insect species had been reported as quinoa feeders within the Chilean territory. Specifically, the general compendium of insect pests of economic importance in Chile reported one aphid (*Smynthurodes betae* Westwood (Hemiptera: Aphididae)), one chinchbug (*O. miles*), and three Lepidoptera species (*Helicoverpa zea* (Boddie), *Rachiplusia nu* Guenée, and *S. recurvalis*) [10]. Another study that focused on caterpillars reported five Lepidoptera species found in quinoa in central Chile, including *E. media*, *Copitarsia turbata* (Herrich-Schäffer), *R. nu*, *C. versurella,* and *A. similalis* [11]. The most recent compendium of arthropod species found in Chilean agricultural plants reported a total of 8 taxa, including *Copitarsia* sp., *H. zea*, *O. miles*, *R. nu*, *Sigelgaita chilensis* Heinrich, *S. betae*, *S. recurvalis*, and *Tapajosa* sp. [12]. However, we found a few incorrect species names and host use attributions in Klein-Koch and Waterhouse [12] and made appropriate corrections to construct Table 1. Specifically, *Sigelgaita chilensis* Heinrich does not feed on quinoa [10]; thus, it was not included here. Additionally, Klein-Koch and Waterhouse [12] lists *Tapajosa* sp. as a quinoa feeder, but as stated by Logarzo et al. [13], *Tapajosa* sp. was identified later as *A. centrolinea*. Finally, there is uncertainty about the identity of the species reported as *C. turbata* [11] because there is considerable confusion among *Copitarsia* species in South American literature [34]. Therefore, all individuals identified as *Copitarsia* during the conduction of this study are reported here as *Copitarsia* spp. and were saved for further taxonomic analysis.

It is possible that other insect species also feed on quinoa in Chile, but for which further studies are needed to clarify its potential presence on quinoas in Chile. For instance, this is the case for the genera *Rhinacloa* (Miridae), *Xenogenus* (Rhopalidae), *Empoasca* (Cicadellidae), *Bergallia* (Cicadellidae), *Conoderus* (Elateridae), *Cylydrorhinus* (formerly = *Adioristus*) (Curculionidae), *Tetraonyx* (Meloidae), and *Symmetrischema* (Gelechiidae). Specifically, regarding *Rhinacloa* sp. reported from the Altiplano area [7,16], there are five species in Chile, including *R. aricana* Carvalho, *R. azapa* Schuh and Schwartz, *R. incaicus* (Carvalho and Gomes), *R. penai* Schuh and Schwartz, and *R. peruana* Schuh and Schwartz [22]. The species *Xenogenus picturatum* Berg. was reported on quinoa [5,15], but only *Xenogenus gracilis* (Reed) is present in Chile [10]. Likewise, the genera *Empoasca* and *Bergallia* (Cicadellidae) have been reported attacking quinoa in Perú [35], for which *Empoasca curveola* Oman [10] and *Bergallia valdiviana* Berg [36] are found in Chile, but we did not find evidence of their association with the cultivation of quinoa in Chile. Similarly, Dughetti [15] and Cruces et al. [5] also report *Conoderus* sp. on quinoa; however, we did not find either *Conoderus chilensis* (Schwartz) or *C. rufangulus* (Gyllenhal), which are the two species present in Chile [10]. Another taxon previously reported only at the level of genus is *Adioristus* sp. [5,7,16], but this name is a synonym of *Cylydrorhinus,* as stated by Wibmer and O’Brien [37]. Valoy et al. [38] reported *Tetraonyx* sp. with no details about species identity. For this genus in Chile, Elgueta and Arriagada [39] reported *Tetraonyx limbata*, *T. parviceps* and *T. septemguttata*, however there is no information that suggests quinoa as part of their host range. Regarding another potential quinoa pest, Dughetti [15] and Cruces et al. [5] also reported *Symmetrischema* sp., for which *S. nanum* Povolný, *S. striatella* (Murtfeldt), and *S. tangolias* (Gyen) are in Chile [26,40,41]. Undoubtedly, future samplings and taxonomic studies could expand the list of species reported in this study.

Most species that feed on quinoa are chewing stages belonging to Lepidoptera and a few species of Coleoptera and Orthoptera, followed by piercing–sucking Hemiptera. Insects with a chewing feeding habit feed on leaves, inflorescences, and developing grains. Flower- and seed-feeding insects, such as Gelechiidae and Noctuidae, often cause serious damage to quinoa production [5,7,16]. Piercing–sucking hemipterans, such as Aphididae and Cicadellidae, feed on phloem/xylem sap extracted from leaves, stems, and inflorescences, while chinch bugs in Heteroptera may also feed on immature grains [15]. In contrast, Thysanoptera species can puncture and extract cell contents of leaves, buds, inflorescences, and pollen. Agromyzidae species use a different feeding strategy, as the larvae of *L. huidobrensis* construct feeding galleries in the leaves of quinoa, and adult females puncture the leaves with their ovipositor to feed on cell content [5]. The larvae of a few species feed underground, such as *Epitrix* and those belonging to Anthomyiidae [5,18]. 

Although a variety of insects feed on quinoa, some are rarely seen in the field [9]. Additionally, only a few species are commonly observed in high population numbers, thereby causing concerns to growers about potential yield losses [5,7,9]. Typically, *Eurysacca melanocampta* (Meyrick) and *E. quinoae* are frequently cited as the most significant quinoa pests in Perú and Bolivia [5,7]. Nonetheless, the geographical location of quinoa production has been shown to influence species richness and its abundance, even within the same country. In a recent study conducted in two lowland sites (La Molina and Majes) and one highland site (San Lorenzo) of Perú, Cruces et al. [9] detected higher populations of *M. euphorbiae*, *E. melanocampta*, and *L. huidobrensis* in La Molina, as well as of *F. occidentalis* in the locality of Majes; only *E. melanocampta* was a major pest in San Lorenzo. Concordantly, we identified four distinct groups of species associated with quinoa along a latitudinal gradient of Chile (Table 2). Nine unique insect species feed on quinoa in the northern territory (Cluster 1), including *A. experta*, *A. centrolinea*, *C. includens*, *E. quinoae, Pseudomeloe* sp., *S. frugiperda*, *S. ochrea*, *S. recurvalis,* and *T. rubricollis*. This geographic area is close to the borders with Perú and Bolivia, and it is geographically isolated from the central and southern quinoa production areas by the Atacama Desert. Indeed, many of these species are also reported from the Altiplano areas of Bolivia and Perú [5]. During our observations in the quinoas grown in the altiplano area of Ancovinto, we found relatively high numbers of *E. quinoae*, *H. atacamae*, *H. gelotopoeon*, and *Copitarsia* sp. larvae feeding on leaves, flowers, and developing grains, as well as occasional clusters of the aphid *A. craccivora* during grain development, but otherwise, other species were uncommon. 

Coastal ecotypes grown at higher latitudes are potentially affected by a distinct assemblage of insect species. Particularly, the central region (Cluster 3) concentrates the highest number of species, with unique species including *E. media*, *Epitrix* sp., *O. flavosparsus*, and *C. versurella*. Nonetheless, not all the species present in this area have been signified as major pests in other lowland areas of quinoa production [9]. Indeed, during our field studies, we only observed population outbreaks of *Copitarsia* sp., *L.* *lineatoventris*, and *O. flavosparsus*, as well as occasional infestations with *A. craccivora* and *L. huidobrensis*. In the southernmost production area (Cluster 4), the number of herbivorous arthropod species is the lowest, therefore representing potential advantages for the sustainable production of quinoa at higher latitudes. 

Most species potentially found on quinoa in Chile show a wide range of host use. Generalist herbivores are often major pests in other agricultural crops, and therefore, they could potentially colonize quinoas grown near vegetables and other crop species as quinoa production areas diversify outside the highlands of the Andes. This has been reported in Perú, where lowland quinoas are negatively affected by polyphagous feeders such as *F. occidentalis*, *M. euphorbiae,* and *L. huidobrensis* [9]. Similarly, several polyphagous insect species negatively affect quinoas in other countries, such as Argentina [15], the United States [18], and Italy [42,43,44]. Interestingly, many of these generalist feeders are also cosmopolitan invasive pests that represent potential pest problems in other areas of the world where quinoa production is expanding.

## 4. Materials and Methods

### 4.1. Field Sampling and Species Identification

Commercial and experimental quinoa plantations were sampled periodically between 2015 and 2018 in 7 sampling sites within the 4 political regions named (abbr.) Tarapacá, Metropolitana, O’Higgins, and Los Lagos. For each region, sampling details are provided below.

- Tarapacá

Ancovinto site with commercial plantations of salares ecotype (20 ha) (19°24′ S, 68°35′ W, 3720 m.a.s.l.). Inspected: 10 December 2016, 27 January 2017, 7 April 2017, and 29 January 2018.

- Metropolitana

Pirque site. Research facility with experimental plantations (1 ha) of coastal ecotype (33°40′ S, 70°35′ W, 653 m.a.s.l.). Inspected: 22 December 2015, 14 January 2016, and 10 October 2018.
Santiago site, research facility with demonstrative plantation of coastal ecotype (33°29′ S, 70°36′ W, 576 m.a.s.l.). Inspected on a monthly basis from November through April of 2016, 2017, and 2018.

- O’Higgins

Cahuil site with commercial plantation of coastal ecotype (10 ha) (34°29′, 72°01′ W, 40 m.a.s.l.). Inspected: 12 October 2016, 12 December 2016, 12 January 2016, and 22 January 2017.
Pailimo site with commercial plantation of coastal ecotype (5 ha) (34°15′ S, 71°47′ W, 242 m.a.s.l.). Inspected: 12 October 2016, 12 January 2016, and 22 January 2017.

- Los Lagos

Ancud sites 1 and 2 with commercial plantations of coastal ecotypes (0.1 and 0.2 ha) (41°50′ S, 74°00′ W, 7 m.a.s.l., and 42°00′ S, 73°53′ W, 38 m.a.s.l.). Inspected: 16 December 2016, 13 January 2017, and 3 February 2017.

In these sites, quinoa plants were scouted by whole-plant visual inspections, plant beating, and using a sweeping net. Similar sampling efforts were devoted to each sampling date, which corresponded to 1 h of scouting. Special attention was given to collecting insects that were actively feeding. For inspections, each site was monitored in a random pattern, selecting at least 5 sectors where 5–10 plants were sampled per sector. Plant tissues affected by each species were annotated as leaves, stems, and/or panicles (flowers and/or seeds). Immature stages were brought to the laboratory and reared individually until adult emergence. Identifications of field-collected specimens were conducted on mounted adult specimens and comparing their morphological traits with available taxonomic publications [10,23,27,28,30,31,32,33,45,46,47], or directly with identified specimens in the Colección Nacional de Insectos, Museo Nacional de Historia Natural, Santiago, Chile (MNNC). Voucher specimens are conserved in the Entomological Collection of the Museo Nacional de Historia Natural, Santiago, Chile and in the Entomological Collection of Facultad de Agronomía e Ingeniería Forestal, Pontificia Universidad Católica de Chile, Santiago. 

### 4.2. Total Number of Expected Species 

First, we compiled records of insect and mite species to create a comprehensive list of arthropods reported to feed on quinoa around the world. We used the following scientific articles, books, and technical reports: [5,7,10,11,15,16,18,38,41,48]. Thereafter, this list was checked for the correct use of scientific names and updated. Arthropod species names were checked against currently available records for species present in Chile and updated with the species found in the surveys conducted in this study. 

This checklist of arthropods associated with quinoa production in Chile was supplemented with bibliographical information about their geographical distribution in Chile, as well as their feeding habits and host range. Feeding habits were classified according to their mouthparts into chewing, piercing–sucking, cell-puncturing, and leaf-mining habits. Host range use was classified either as generalist or specialist if the species had been reported feeding on several non-related plant genera or only on a few related plant species [49]. Additionally, the species were classified according to their geographical origin into Nearctic (North America), Neotropic (Central and South America), Nearctic—Neotropic (American continent in general), Palearctic (Eurasia), Indo-Malayan (India—Asia), Chile (Native), or as unknown [10,13,20,27,28,50].

### 4.3. Data Analysis 

Multivariate cluster analysis was conducted to find associations among species according to their presence/absence within each political region of Chile (latitudinal variables). Data matrix was constructed using the presence or absence (values 1 or 0, respectively) of the 43 arthropod taxa (reported in Table 1) in each of the 14 political regions that represent the variety of environments where quinoa is produced in Chile, specifically: Región de Arica y Parinacota (~17.5–19.1° S), Región de Tarapacá (~19.2–21.6° S), Región de Antofagasta (~21.7–25.8° S), Región de Atacama (~25.9–29.2° S), Región de Coquimbo (~29.3–32° S), Región de Valparaíso (~32.1–33° S), Región Metropolitana de Santiago (~33.1–34° S), Región del Libertador General Bernardo O’Higgins (~34.1–34.8° S), Región del Maule (~34.9–36.2° S), Región de Ñuble (~36.2–36.9° S), Región del Bio-Bio (~36.9–37.8° S), Región de La Araucanía (~37.9–39.5° S), Región de Los Ríos (~39.5–40.4° S), and Región de Los Lagos (~40.4–43.7° S). A dendrogram was constructed by distance correlation coefficient and complete linkage amalgamation steps functions using Minitab 17 software (Minitab Inc., State College, PA, USA). Specifically, for the linkage method, the distance between two clusters was calculated with the furthest-neighbor method, which is the maximum distance between variables of one cluster relative to another cluster. Distance between variables was calculated using the correlation method to consider positively correlated data to be closer than negatively correlated data, as it calculates distances between 0 and 1 for positive correlations and values between 1 and 2 for negative correlations. A total of 13 amalgamation steps were considered to create the dendrogram. Similarity levels ranged between 100 (13 clusters) and 91.3% (7 clusters) for the first 7 steps, and between 88.2 to 85.8% for steps 8 (6 clusters) to 10 (4 clusters), after which the similarity dropped below 74%.

## Figures and Tables

**Table 1:** Species list and characteristics of herbivore arthropods that use quinoa as a host plant and are present in Chile.

Species	Distribution Range in Chile (° S)	Feeding Habit	Host Range	Geographical Origin	References
Orthoptera: Acrididae					
*Dichroplus maculipennis* (Blanchard, 1851)	29–56	Chewing	Generalist	Neotropic	[5,14,15]
Hemiptera: Cicadellidae					
*Anacuerna centrolinea* (Melichar, 1925)	19–22	Piercing–sucking	Generalist	Neotropic	[5,13]
*Paratanus exitiosus* Beamer, 1943	32–41	Piercing–sucking	Generalist	Neotropic	[5,7,10,16]
Hemiptera: Aphididae					
*Aphis craccivora* Koch, 1854	18–44 ^a,b,c^	Piercing–sucking	Generalist	Palearctic	[5,7,10,15,16]
*Aphis gossypii* Glover, 1877	18–44	Piercing–sucking	Generalist	Unknown	[5,7,12,16,17]
*Macrosiphum euphorbiae* (Thomas, 1878)	18–56 ^a,b,c,d^	Piercing–sucking	Generalist	Neotropic—Nearctic	[5,7,10,16,17,18]
*Myzus persicae* (Sulzer, 1776)	18–56 ^b^	Piercing–sucking	Generalist	Indo-Malayan	[5,7,10,16,17]
*Smynthurodes betae* Westwood, 1849	18–22; 32–56	Piercing–sucking	Generalist	Unknown	[10,17]
Hemiptera: Triozidae					
*Heterotrioza chenopodii* (Reuter, 1876) (=*Trioza chenopodii* Reuter)	29–34	Piercing–sucking	Specialist	Palearctic	[5,15,19]
Hemiptera: Pentatomidae					
*Nezara viridula* (Linnaeus, 1758)	18–56	Piercing–sucking	Generalist	Unknown	[5,10,15]
Hemiptera: Lygaeidae					
*Nysius simulans* (Stål, 1859)	29–38	Piercing–sucking	Generalist	Neotropic	[10]
*Oncopeltus miles* (Blanchard, 1852)	19–40	Piercing–sucking	Specialist	Chile	[10,20]
Hemiptera: Coreidae					
*Leptoglossus chilensis* (Spinola, 1852)	26–44	Piercing–sucking	Generalist	Neotropic	[5,15,21]
Hemiptera: Rhopalidae					
*Liorhyssus hyalinus* (Fabricius, 1794)	18–38	Piercing–sucking	Generalist	Unknown	[10]
*Liorhyssus lineatoventris* (Spinola, 1852)	30–40 ^b^	Piercing–sucking	Generalist	Neotropic	[22]
Hemiptera: Miridae					
*Orthotylus flavosparsus* (Sahlberg, 1841)	33–34 ^b^	Piercing–sucking	Generalist	Unknown	[5,15,22]
Thysanoptera: Thripidae					
*Frankliniella occidentalis* (Pergande, 1895)	18–44 ^b,c^	Cell puncturing	Generalist	Nearctic	[12,18]
*Thrips tabaci* Lindeman, 1889	18–40	Cell puncturing	Generalist	Unknown	[7,10,16,18]
Diptera: Agromyzidae					
*Liriomyza huidobrensis* Blanchard, 1926	18–49 ^a,b,c^	Leaf mining	Generalist	Neotropic	[5,7,10,16]
Coleoptera: Chrysomelidae					
*Epitrix* sp.	32–34 ^b^	Chewing	Unknown	Neotropic—Nearctic	[5,7,10,12,16]
Coleoptera: Curculionidae					
*Trichocyphus rubricollis* (Blanchard, 1847)	18–26 ^a^	Chewing	Unknown	Neotropic	[23]
Coleoptera: Meloidae					
*Pseudomeloe* sp.	19–22 ^a^	Chewing	Unknown	Neotropic	[7]
Lepidoptera: Coleophoridae					
*Coleophora versurella* Zeller, 1849	32–40	Chewing	Specialist	Neotropic—Nearctic	[11,24,25]
Lepidoptera: Crambidae					
*Achyra similalis* (Guenée, 1854) (=*Loxostege similalis* (Guenée))	18–40 ^b^	Chewing	Specialist	Neotropic—Nearctic	[10,11,25]
*Spoladea recurvalis* (Fabricius, 1794)	18–19	Chewing	Specialist	Neotropic	[5,7,10]
Lepidoptera: Gelechiidae					
*Eurysacca media* Povolný, 1986	32–34	Chewing	Specialist	Neotropic	[11,26,27]
*Eurysacca quinoae* Povolný, 1997	19 ^a^	Chewing	Specialist	Neotropic	[28]
Lepidoptera: Noctuidae					
*Agrotis experta* (Walker, 1869) (=*Feltia experta* (Walker))	18–26	Chewing	Generalist	Neotropic	[5,7,10,16]
*Agrotis ipsilon* (Hufnagel, 1766)	18–44 ^c^	Chewing	Generalist	Unknown	[5,7,10,16]
*Agrotis malefida* (Guenée, 1852)	32–56	Chewing	Generalist	Neotropic	[7,10]
*Copitarsia* spp. (species complex)	18–56 ^a,b,c,d^	Chewing	Generalist	Neotropic	[5,10,11,12,16,29]
*Chrysodeixis includens* (Walker, 1858) (=*Pseudoplusia includens* (Walker)) (=*Phytometra oo* (Cramer))	18–26	Chewing	Generalist	Neotropic—Nearctic	[5,10,12]
*Feltia subterranea* (Fabricius, 1794) (=*Agrotis subterranea*)	18–40 ^b,c^	Chewing	Generalist	Neotropic—Nearctic	[5,10]
*Helicoverpa atacamae* Hardwick, 1965	18–41 ^a^	Chewing	Generalist	Neotropic	[5,16,30]
*Helicoverpa gelotopoeon* (Dyar, 1921)	18–40 ^a^	Chewing	Generalist	Neotropic	[5,12,15,30]
*Helicoverpa zea* (Boddie, 1850)	18–44	Chewing	Generalist	Neotropic—Nearctic	[5,7,10,12,16,18]
*Peridroma saucia* (Hübner, 1808)	18–56	Chewing	Generalist	Unknown	[5,7,10,16]
*Rachiplusia nu* (Guenée, 1852)	18–44	Chewing	Generalist	Neotropic—Nearctic	[5,10,11,15]
*Spodoptera eridania* (Stoll, 1782)	18–33	Chewing	Generalist	Neotropic—Nearctic	[5,7,10,16]
*Spodoptera frugiperda* (J.E. Smith, 1797)	18–22	Chewing	Generalist	Neotropic—Nearctic	[5,7,10,15,16]
*Spodoptera ochrea* (Hampson, 1909)	18–22	Chewing	Generalist	Neotropic	[5,10]
*Trichoplusia ni* (Hübner, 1803)	18–44 ^b^	Chewing	Generalist	Unknown	[12,18]
Acari: Tetranychidae					
*Tetranychus urticae* Koch, 1836	18–44 ^b^	Cell puncturing	Generalist	Unknown	[5,10]

^a^ Collected in this study in Tarapacá (19°24′ S, 68°35′ W); ^b^ Metropolitana (33°40′ S, 70°35′ W, or 33°29′ S, 70°36′ W); ^c^ O’Higgins (34°29′, 72°01′ W, or 34°15′ S, 71°47′ W); ^d^ Los Lagos (41°50′ S, 74°00′ W, or 42°00′ S, 73°53′ W).

**Table 2:** Description of the clustering patterns of arthropod species associated with quinoa in Chile.

Cluster Group	Geographical Limits of Each Cluster (° S)	Species Present within the Delimited Area *
1	17.5–25.8	**32 species:***Achyra similalis*, *Agrotis experta*, *Agrotis ipsilon*, *Anacuerna centrolinea*, *Aphis craccivora*, *Aphis gossypii*, *Chrysodeixis includens*, *Copitarsia* spp., *Eurysacca quinoae*, *Feltia subterranea*, *Frankliniella occidentalis*, *Helicoverpa atacamae*, *Helicoverpa gelotopoeon*, *Helicoverpa zea*, *Liorhyssus hialinus*, *Liriomyza huidobrensis*, *Macrosiphum euphorbiae*, *Myzus persicae*, *Nezara viridula*, *Oncopeltus miles*, *Peridroma saucia*, *Pseudomeloe* sp., *Rachiplusia nu*, *Smynthurodes betae*, *Spodoptera eridania*, *Spodoptera frugiperda*, *Spodoptera ochrea*, *Spoladea recurvalis*, *Tetranychus urticae*, *Thrips tabaci, Trichocyphus rubricollis*, *Trichoplusia ni*
2	25.9—32.0	**27 species:***Achyra similalis*, *Agrotis ipsilon*, *Aphis craccivora*, *Aphis gossypii*, *Copitarsia* spp., *Dichroplus maculipennis, Feltia subterranea, Frankliniella occidentalis, Helicoverpa atacamae, Helicoverpa gelotopoeon, Helicoverpa zea, Heterotrioza chenopodii, Leptoglossus chilensis*, *Liorhyssus hialinus*, *Liorhyssus lineatoventris*, *Liriomyza huidobrensis*, *Macrosiphum euphorbiae*, *Myzus persicae*, *Nezara viridula*, *Nysius simulans*, *Oncopeltus miles*, *Peridroma saucia*, *Rachiplusia nu*, *Spodoptera eridania*, *Tetranychus urticae*, *Thrips tabaci*, *Trichoplusia ni*
3	32.1—39.5	**34 species:***Achyra similalis*, *Agrotis ipsilon*, *Agrotis malefida*, *Aphis craccivora*, *Aphis gossypii*, *Coleophora versurella*, *Copitarsia* spp., *Dichroplus maculipennis*, *Epitrix* sp., *Eurysacca media*, *Feltia subterranea*, *Frankliniella occidentalis*, *Helicoverpa atacamae*, *Helicoverpa gelotopoeon*, *Helicoverpa zea*, *Heterotrioza chenopodii*, *Leptoglossus chilensis*, *Liorhyssus hialinus*, *Liorhyssus lineatoventris*, *Liriomyza huidobrensis*, *Macrosiphum euphorbiae*, *Myzus persicae*, *Nezara viridula*, *Nysius simulans*, *Oncopeltus miles*, *Orthotylus flavosparsus*, *Paratanus exitiosus*, *Peridroma saucia*, *Rachiplusia nu*, *Smynthurodes betae*, *Spodoptera eridania*, *Tetranychus urticae*, *Thrips tabaci*, *Trichoplusia ni*
4	39.5—43.7	**22 species:***Achyra similalis*, *Agrotis ipsilon*, *Agrotis malefida*, *Aphis craccivora*, *Aphis gossypii*, *Copitarsia* spp., *Dichroplus maculipennis*, *Frankliniella occidentalis*, *Helicoverpa atacamae*, *Helicoverpa zea*, *Leptoglossus chilensis*, *Liorhyssus lineatoventris*, *Liriomyza huidobrensis*, *Macrosiphum euphorbiae*, *Myzus persicae*, *Nezara viridula*, *Paratanus exitiosus*, *Peridroma saucia*, *Rachiplusia nu*, *Smynthurodes betae*, *Tetranychus urticae*, *Trichoplusia ni*

* For information regarding species-specific distribution within each cluster, see Table 1.

## Data Availability

Data is contained within the article.
